# Interface Hepatitis over Grade 2 May Differentiate Chronic Inflammation Associated with CHB from NAFLD in the Early Stage

**DOI:** 10.1155/2020/3584568

**Published:** 2020-04-20

**Authors:** Yong-fen Zhu, Jin Wang, Jia-zhui Fang, Qiao Yang, Fang-fang Lv

**Affiliations:** Department of Hepatology and Infection, Sir Run Run Shaw Hospital, Affiliated with School of Medicine, Zhejiang University, Hangzhou 310016, China

## Abstract

**Background:**

Patients with chronic hepatitis B (CHB) concomitant with nonalcoholic fatty liver disease (NAFLD) are increasing.

**Objectives:**

To identify pathological features that can be used to differentiate between chronic inflammation caused by CHB and that caused by NAFLD.

**Methods:**

Patients with CHB (*n* = 31) needing antiviral treatment, NAFLD (*n* = 50), or CHB-NAFLD (*n* = 51) who underwent biopsy were retrospectively enrolled. Pathological characteristics of chronic inflammation were evaluated using the METAVIR scoring system. The rates of three pathological characteristics were first compared in patients with NAFLD and those with CHB, then compared after fibrosis matching, and were finally compared in CHB-NAFLD patients with different viral loads.

**Results:**

The rates of interface hepatitis over grade 2 and fibrosis over grade 2 were significantly higher in the CHB group than in the NAFLD group (100% *vs*. 4% and 80.6% *vs*. 22%; both *P* < 0.0001), while no significant difference was observed in the rate of lobular inflammation over grade 2 between the two groups. After fibrosis matching, in patients with F0–2 fibrosis, the rate of interface hepatitis over grade 2 in CHB was significantly higher than that in NAFLD (100% *vs.* 0%; *P* < 0.0001). In CHB-NAFLD patients with F0–2 fibrosis, the rate of interface hepatitis over grade 2 in cases with a high viral load was significantly higher than cases with a low viral load (66.6% *vs.* 0%; *P* < 0.0001). The rate of lobular inflammation showed no difference between groups.

**Conclusion:**

Interface hepatitis over grade 2 can be used for the differential diagnosis of chronic inflammation associated with CHB or NAFLD in the early stage.

## 1. Introduction

Hepatitis B virus (HBV) infection is one of the major causes of chronic liver disease, which ranges from chronic hepatitis to liver cirrhosis and even hepatocellular carcinoma [1, 2]. It is estimated that HBV infection afflicts approximately 240 million patients with chronic hepatitis B (CHB) worldwide [3]. CHB is most prevalent in China, which has about 90 million patients with this disease [4]. Nonalcoholic fatty liver disease (NAFLD), another important cause of chronic liver disease, is characterized by fat deposition in hepatocytes ranging from simple steatosis to nonalcoholic steatohepatitis (NASH) and NASH-related cirrhosis [5]. The prevalence of NAFLD ranges from 7.9% to 54.0% in Asia, with 20.1% cases in China [5]. NAFLD is becoming one of the most common liver diseases worldwide [6, 7].

With the growing epidemic of NAFLD, an increasing number of patients reportedly have CHB concomitant with NAFLD [8–10]. The prevalence of NAFLD in CHB is about 13.5–19% [11]. Chronic inflammation with persistently elevated transaminase levels can occur in both NAFLD and CHB. In CHB patients with NAFLD, chronic hepatitis may be caused by CHB, NAFLD, or both [12, 13]. A clinical study of antiviral therapy in CHB patients with NAFLD found that when NAFLD patients were diagnosed with liver biopsy and treated with interferon, NAFLD did not affect the efficacy of antiviral therapy, whereas when NAFLD patients were diagnosed with Doppler ultrasound and treated with nucleoside analogues, NAFLD affected the efficacy of antiviral therapy, resulting in poor biochemical and virological response [1]. What causes the difference in the treatment outcome, the antiviral treatment regimen (interferon *vs.* nucleoside analogues), or the different methods assessing chronic inflammation (liver biopsy *vs.* alanine aminotransferase (ALT) and Doppler ultrasound) remains unclear. Doppler ultrasound and abnormal ALT cannot be used for the assessment of the etiology of chronic inflammation. Some researchers have speculated that the differences may be due to NAFLD-induced elevated transaminase being misdiagnosed as CHB for antiviral therapy [14].

CHB is pathologically characterized by inflammation in the portal area and the surrounding area. Determining whether or not patients should receive antiviral treatment is based on the severity of inflammation and fibrosis, which are graded using the METAVIR scoring system [15, 16]. The METAVIR score composes of the degree of interface hepatitis, lobular inflammation, and portal fibrosis and can be applied for the grading of inflammation and fibrosis for deciding further antiviral treatment. NAFLD is pathologically characterized by lobular inflammation and balloon-like degeneration, which is assessed using the NAFLD activity score (NAS) [17]. When CHB combined with NAFLD occurs, chronic inflammation caused by CHB is characterized by more severe inflammation in the portal area [11]. However, there have been no reports about the detailed pathological differences between CHB with and without NAFLD.

Generally, patients with chronic inflammation caused by NAFLD are subjected to lifestyle intervention, whereas patients with CHB are subjected to anti-HBV treatment [18, 19]. Therefore, when treating chronic inflammation in patients with CHB complicated with NAFLD, the etiology of chronic inflammation is important for determining the appropriate treatment, especially antiviral therapy for CHB, because inappropriate anti-HBV treatment results in the economic burden of long-term medication and long-term drug safety.

This study analyzed the pathological features of chronic inflammation of NAFLD and CHB, which are needed for antiviral treatment, to identify the pathological features associated with chronic inflammation and to differentiate CHB from NAFLD. We also confirmed the pathological features in patients with CHB combined with NAFLD and different viral loads.

## 2. Materials and Methods

### 2.1. Patients

Patients who underwent liver biopsy for liver pathology between January 2017 and December 2019 in Sir Run Run Shaw Hospital (Zhejiang, China) were enrolled. Patients with CHB requiring antiviral treatment, NAFLD, or CHB combined with NAFLD were included. Patients with NAFLD were diagnosed as follows [19–21]: had persistent liver function abnormalities for more than 6 months; ultrasound showing fatty liver; liver biopsy showing hepatic steatosis > 5%; and no history of drinking, medication, and viral hepatitis. Patients with CHB were diagnosed as follows [22, 23]: hepatitis B surface antigen- (HBsAg-) positive for 6 months or more; hepatitis B e antigen- (HBeAg-) positive and HBVDNA > 20000 IU/mL or HBeAg-negative and HBVDNA > 2000 IU/mL; ultrasound showing no fatty liver; liver biopsy showing hepatic steatosis > 5%; needed antiviral treatment according to METAVIR scores; and antiviral treatment naïve. Patients with CHB combined with NAFLD were diagnosed as follows: repeated abnormal liver function; HBsAg-positive for 6 months; liver biopsy showing hepatic steatosis > 5%; and no alcoholic liver disease or immune liver disease. Information including gender, age, body mass index (BMI), alanine aminotransferase (ALT), aspartate aminotransferase (AST), rate of HBeAg positivity, hypertension, diabetes, and hyperlipidemia was retrospectively collected from the electronic case records. The study protocol was approved by the Ethics Committee of Sir Run Run Shaw Hospital. Each participant provided written informed consent.

### 2.2. Liver Biopsy and Histopathology Assessment

Liver tissue with a length of about 1.0–2.0 cm was obtained using a 16G puncture needle through vacuum suction under ultrasonic localization. The tissue was fixed in 4% dehydrated, embedded in paraffin, and sectioned continuously. The slides were subjected to hematoxylin and eosin (H&E) staining and silver staining of the reticular fiber and then read by an experienced pathologist. The METAVIR scoring system is the standard semiquantitative pathology criteria for whether antiviral treatment is needed for CHB patients. It includes the scores of interface hepatitis, lobular inflammation, and portal fibrosis. Therefore, in the present study, interface hepatitis, lobular inflammation, and portal fibrosis were assessed using the METAVIR scoring system [15], and steatosis was evaluated using the NAS scoring system [17]. Liver histology was defined as follows. Interface hepatitis (also known as piecemeal necrosis) was divided into four grades (0–3): 0, absent; 1, local inflammation of the periportal plate in some portal tract; 2,diffuse alteration of the periportal plate in some portal tract or focal lesions around all portal areas; and 3, diffuse alteration of the periportal plate in all portal areas. Lobular inflammation was divided into three grades (0–2): 0, one focus or less per hepatic lobule; 1, at least 1 focus per hepatic lobule; and 2, several foci per hepatic lobule or bridging necrosis and fusion necrosis. Fibrosis was divided into five grades (F0–4): 0, absent; 1, portal fibrosis without septa; 2, portal fibrosis with rare septa; 3, numerous septa without cirrhosis; and 4, cirrhosis. Steatosis was divided into four grades (S0–3): 0, <5%; 1, 5–33% (including 33); 2, 33–66%; and 3, >66%.

### 2.3. Statistical Analysis

Results are presented as medians (interquartile range) for continuous variables and percentage values for categorical variables. Statistical analysis was performed using SPSS 17.0 for Windows (SPSS, Inc., Chicago, IL, USA). *P* values were determined by Fisher's exact test for categorical variables and Wilcoxon rank-sum test for continuous variables. *P* < 0.05 was considered statistically significant.

## 3. Results

### 3.1. Baseline Characteristics of the Participants

A total of 132 participants were included, with 50, 31, and 51 cases of NAFLD, CHB, and CHB combined with NAFLD, respectively. The baseline characteristics of the patients are shown in Table 1. Significant differences were observed among the three groups in BMI (27.99 ± 3.19 vs. 21.58 ± 2.38 vs. 25.62 ± 3.45, *P* < 0.0001) and transaminase levels (ALT 117.78 ± 84.17 vs. 68.35 ± 68.01 vs. 62.25 ± 50.80, *P* < 0.0001; AST 56.20 ± 31.81 vs. 42.32 ± 36.26 vs. 35.80 ± 20.15, *P* = 0.001). The rate of hyperlipidemia in the NAFLD group was higher than that in the CHB combined with the NAFLD group (46/50 vs. 24/51, *P* < 0.0001). Similarly, hepatic steatosis was more severe in the NAFLD group.

### 3.2. Comparison of Pathological Features between CHB and NAFLD

As shown in Table 2, the rate of interface hepatitis over grade 2 was significantly higher in the CHB group than in the NAFLD group (100% (31/31) vs. 4% (2/50); *P* < 0.0001). The rate of portal fibrosis over grade 2 was also significantly higher in the CHB group than in the NAFLD group (80.6% (25/31) in the CHB group vs. 22% (11/50) in the NAFLD group). No significant difference was observed in the rate of lobular inflammation over grade 2 between the two groups (41.9% (13/31) vs. 24% (12/50); *P* = 0.091).

### 3.3. Comparison of Pathological Features between CHB and NAFLD after Fibrosis Hierarchical Matching

There were two cases of interface hepatitis over grade 2 in the NAFLD group, and all cases presented with fibrosis over F2. The rates of interface hepatitis and lobular inflammation were compared between the NAFLD and CHB groups in patients with fibrosis less than F2 after fibrosis matching. As shown in Table 3, none of the 45 patients with NAFLD showed interface hepatitis over grade 2, whereas all 23 patients with CHB showed interface hepatitis over grade 2 (*P* < 0.0001). No significant difference was observed in the rate of lobular inflammation between groups.

### 3.4. Pathological Characteristics of Patients with CHB Combined with NAFLD

**Figure 1:**
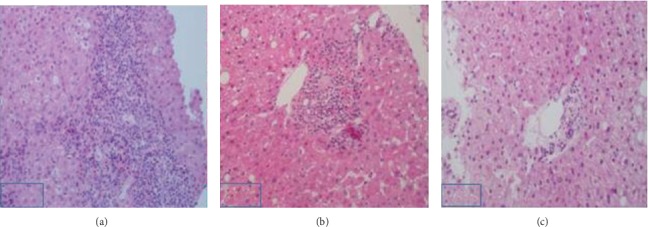
Representative pictures of interfacial hepatitis. (a) Male, 27 years old, chronic HBV infection, ALT 66 U/L, HBV DNA 1.58∗E8 IU/mL; liver biopsy revealed grade 3 interface hepatitis, grade 2 fibrosis, and hepatic steatosis 40–50% (S2), being diagnosed with CHB combined with NAFLD; (b) male, 34 years old, chronic HBV infection, ALT 47 IU/L, HBV DNA E7 IU/mL, liver biopsy revealed grade 1 interface hepatitis, grade 1-2 fibrosis, hepatic steatosis 50–60% (S2), being diagnosed with NAFLD while infected with HBV; (c), Male, 36 years old, chronic HBV infection, HBVDNA 57, ALT 58 U/L; liver biopsy revealed grade 0 interface hepatitis, grade 0 fibrosis, and hepatic steatosis 30% (S1), being diagnosed with NAFLD while infected with HBV.

Patients were divided into two groups according to HBV DNA load, low viral load group (HBVDNA < 2000 IU/mL, *n* = 19) and high viral load group (HBVDNA ≥ 2000 IU/mL, *n* = 21). As shown in Table 4, the rates of interface hepatitis and lobular inflammation were compared between both groups in patients with fibrosis less than F2. The rate of interface hepatitis over grade 2 was 66.6% in the high viral load group, whereas there was no interface hepatitis in the low viral load group (*P* < 0.0001). The rate of lobular inflammation showed no difference between groups. Liver pathology pictures of interface hepatitis in each group are shown in Figure 1.

## 4. Discussion

In the present study, the histopathology features including interface hepatitis, lobular inflammation, and fibrosis were compared between patients with CHB and NAFLD. The features were further compared after fibrosis matching. We found that patients with CHB showed a higher rate of interface hepatitis over grade 2. Further study in patients with CHB combined with NAFLD showed that the rate of interface hepatitis over grade 2 was higher in the high viral load group than in the low viral load group.

Inflammation in the portal area includes inflammatory cell infiltration in the portal area and piecemeal necrosis (interface hepatitis) caused by infiltration of inflammatory cells into the liver cells. The pathological characteristics of CHB are the portal and surrounding inflammation. For patients with CHB, interface hepatitis, lobular inflammation, and portal fibrosis should be evaluated according to the METAVIR scoring system, and patients with inflammation over grade 2 as well as fibrosis over grade 2 should be subjected to antiviral therapy [15, 16, 18]. Inflammation in the portal area has not been evaluated in adult NAFLD. However, lymphocytic infiltration in the portal area is considered to be the pathological feature of progressive NAFLD [24]. Studies about interface hepatitis in NASH are not available yet.

For CHB patients, inflammation over grade 2 and portal fibrosis over grade 2 indicate that the patient has entered the reactivation phase during HBV infection and anti-HBV therapy is required [8, 11]. Thus, the rates of interface hepatitis, lobular inflammation, and fibrosis over grade 2 were compared in the present study. Our data showed that the rates of interface hepatitis over grade 2 and fibrosis over grade 2 were significantly higher in the CHB group than in the NAFLD group, while no significant difference was observed in the rate of lobular inflammation over grade 2 between the two groups. These results suggest that lesions in the portal area of the liver of CHB patients were significantly more severe than those in NAFLD patients, which is consistent with a previous report about the pathological features of CHB and NAFLD [17].

In the NAFLD group, two patients with interface hepatitis over grade 2 both showed significant hepatic fibrosis. A previous study showed that lymphocytic infiltration in the portal area in patients with NAFLD suggests the risk of disease progression [24]. We also observed more severe portal inflammation-interface hepatitis in patients with cirrhosis or precirrhosis, further confirming the above hypothesis that inflammation in the portal area indicates the progression of NAFLD. None of the 45 cases of NAFLD with mild to middle fibrosis (F0–2) showed interface hepatitis over grade 2, whereas all paired 23 cases of CHB with mild to middle fibrosis (F0–2) showed interface hepatitis over grade 2. However, the rate of lobular inflammation was not significantly different between the two groups. Therefore, this study suggests that for patients with early-stage chronic hepatitis (fibrosis ≤ grade 2), interface hepatitis over grade 2 can be used as the pathological feature of diagnosis for differentiating patients with CHB in the immune clearance phase from patients with NAFLD. Interface hepatitis is the typical pathological features of CHB and is the liver damage caused by the activation of the acquired immune T lymphocytes. However, NAFLD is the inflammation caused by the macrophage activation of the natural immune system. The main pathological features of NAFLD are the lobular inflammation and ballooning degeneration of hepatocyte, and the activation of the acquired immune system may also be involved later. Therefore, we speculated that in NAFLD, the activation of acquired immune system and the occurrence of the interface hepatitis and bile duct reaction are intermediate processes promoting liver fibrosis, finally leading to liver cirrhosis [25–27].

The differential diagnostic ability of interface hepatitis over grade 2 CHB and NAFLD was further validated in patients with CHB concomitant with NAFLD. Patients with HBVDNA < 2000 IU/mL reportedly have a low risk of CHB [28]. Thus, these patients with persistent abnormal liver function were divided into high viral load and low viral load groups using 2000 IU/mL as the cutoff value. None of the 19 patients with a low viral load showed interface hepatitis grade 2, suggesting that chronic inflammation in these patients may be caused by NAFLD, which is consistent with a previous study showing that chronic inflammation in patients with CHB and NAFLD coexistent with low viral load may be due to NAFLD [13]. The rate of interface hepatitis over grade 2 in patients with a high viral load was 66.6%, suggesting that chronic inflammation in these patients was mainly because of CHB, and chronic inflammation in the remaining 33.3% of these patients may be caused by NAFLD, and no antiviral treatment is needed. If a liver biopsy is not performed and antiviral treatment is only based on a transaminase abnormality, the 33.3% of patients may be treated using the wrong regimen. Our results explained that if only a transaminase abnormality is used as the indication for antiviral treatment in CHB and NAFLD coexistent patients, some chronic inflammation caused by NAFLD may be wrongly treated by antiviral therapy, resulting in poor response [1].

In the present study, baseline transaminase levels were significantly different among three patient groups. The rates of steatosis in the different grades were also different. These differences were due to the different reasons for biopsy in the different patient groups. CHB patients who were enrolled in this study underwent biopsy because the levels of transaminase were not high enough (twice the upper limit of normal) to receive antiviral treatment, and antiviral treatment was performed after diagnosis using liver pathology. Thus, the transaminase level was low. Patients in the NAFLD group and the CHB combined with the NAFLD group underwent biopsy because of long-term transaminase abnormalities without a clear cause. However, liver pathology was used as the gold standard here, so the difference in baseline transaminase should not affect the results.

There were several limitations in this study. First, this was a single-center study with a small number of patients enrolled. A multicenter study with more patients is needed to further validate the results. Second, this was a cross-sectional study. A cohort study with treatment and follow-up is needed to validate its clinical significance for differential diagnosis.

## 5. Conclusions

In conclusion, by comparing the pathological characteristics of CHB and NAFLD, we found that in chronic liver inflammation at the early stage (F0–2), interface hepatitis over grade 2 can be used as a differential diagnosis feature for CHB and NAFLD, which may provide guidance when treating patients with CHB concomitant NAFLD in the early stage of inflammation.

## Figures and Tables

**Table 1:** Baseline characteristics of the participants.

				*P* value	
	NAFLD (*n* = 50)	CHB (*n* = 31)	CHB and NAFLD (*n* = 51)	NAFLD *vs*. CHB	NAFLD *vs*. CHB and NAFLD
Gender (female/male)	13/37	8/23	8/43	0.985	0.202
Age	37.72 ± 10.49	38.90 ± 7.49	41.29 ± 10.46	0.363	0.115
BMI	27.99 ± 3.19	21.58 ± 2.38	25.62 ± 3.45	<0.0001	<0.0001
ALT	117.78 ± 84.17	68.35 ± 68.01	62.25 ± 50.80	<0.0001	<0.0001
AST	56.20 ± 31.81	42.32 ± 36.26	35.80 ± 20.15	0.001	<0.0001
HBeAg-positive rate	/	15/31	19/51	0.321	/
Hypertension	7/50	/	9/51	/	0.616
Diabetes	6/50	/	6/51	/	0.971
Hyperlipidemia	46/50	/	24/51	/	<0.0001
Steatosis				/	<0.0001
S1	17/50	/	37/51		
S2	27/50	/	13/51		
S3	6/50	/	1/51		

NAFLD: nonalcoholic fatty liver disease; CHB: chronic hepatitis B; BMI: body mass index; ALT: alanine aminotransferase; AST: aspartate aminotransferase; HBeAg: HBV e antigen.

**Table 2:** Inflammatory histopathology of CHB and NAFLD.

	NAFLD (*n* = 50)	CHB (*n* = 31)	*P* value
Interface hepatitis			<0.0001
Grade 0	46 (92%)	0	
Grade 1	2 (4%)	0	
Grade 2	2 (4%)	27 (87.1%)	
Grade 3	0	4 (12.9%)	
Lobular inflammation			0.091
Grade (0, 1)	38 (76%)	18 (58.1%)	
Grade (2)	12 (24%)	13 (41.9%	
Fibrosis			<0.0001
Grade 0	22 (44%)	0	
Grade 1	17 (34%)	6 (19.4%)	
Grade 2	6 (12%)	17 (54.8%)	
Grade 3	5 (10%)	7 (22.6%)	
Grade 4	0	1 (3.2%)	

**Table 3:** Inflammatory histopathology of CHB and NAFLD after fibrosis hierarchical matching.

	1	2	3	*P* value
	NAFLD with severe fibrosis (F3-4, *n* = 5)	NAFLD with mild-moderate fibrosis (F0-2, *n* = 45)	CHB with mild-moderate fibrosis (F0-2, *N* = 23)	2 vs. 3
Interface hepatitis				<0.0001
Grade 0	3 (60%)	43 (95.6%)	0	
Grade 1	0	2 (3.7%)	0	
Grade 2	2 (40%)	0	21 (91.3%)	
Grade 3	0	0	2 (8.7%)	
Lobular inflammation				0.296
Grade (0, 1)	3 (60%)	33 (73.3%)	14 (60.9%)	
Grade 2	2 (40%)	12 (26.7%)	9 (39.1%)	

**Table 4:** Inflammatory histopathology of CHB combined with NAFLD CHB in patients with different vial loads.

	HBV DNA > E4 IU/mL, mild-moderate fibrosis F0-2 (*n* = 21)	HBV DNA < E4 IU/mL, mild-moderate fibrosis F0-2 (*n* = 19)	*P* value
Interface hepatitis			<0.0001
Grade 0	3 (14.3%)	16 (84.2%)	
Grade 1	4 (19%)	3 (15.8%)	
Grade 2	12 (57.1%)	0	
Grade 3	2 (9.5%)	0	
Lobular inflammation			0.246
Grade (0, 1)	13 (61.9%)	15 (78.9%)	
Grade 2	8 (38.1%)	4 (21.1%)	

## Data Availability

The data sets generated and analyzed during the present study are available from the corresponding author on reasonable request.

## Abbreviations

HBV: Hepatitis B virus
CHB: Chronic hepatitis B
NAFLD: Nonalcoholic fatty liver disease
NASH: Nonalcoholic steatohepatitis
NAS: NAFLD activity score.
